# Subcutaneous anakinra in the management of refractory MIS-C in France

**DOI:** 10.3389/fped.2024.1270878

**Published:** 2024-02-23

**Authors:** Perrine Dusser, Alexandre Belot, Fanny Bajolle, Charlotte Kevorkian-Verguet, Ulrich Meinzer, Frédéric Huet, Soizic Tiriau, Isabelle Kone-paut

**Affiliations:** ^1^ CEREMAIA, Pediatric Rheumatology, Bicêtre University Hospital, Assistance Publique-Hôpitaux de Paris, University of Paris Saclay, Le Kremlin Bicêtre, France; ^2^ Pediatric Nephrology, Rheumatology, Dermatology, Reference Centre of Inflammatory Rheumatism and Rare Autoimmune Diseases in Children (RAISE), Hôpital Femme Mère-Enfant, Hospices Civils de Lyon, Bron, France; ^3^ Assistance Publique-Hôpitaux de Paris, M3C Department, Necker-Enfants Malades University Hospital, Université de Paris, Paris, France; ^4^ Department of General Pediatrics, Centre Hospitalier Universitaire de Grenoble, Grenoble, France; ^5^ Department of General Pediatrics, Pediatric Internal Medicine, Rheumatology and Infectious Diseases, National Reference Centre for Rare Pediatric Inflammatory Rheumatisms and Systemic Autoimmune Diseases (RAISE), Robert-Debré University Hospital, Assistance Publique-Hôpitaux de Paris, Paris, France; ^6^ Université Paris Cité, INSERM, Centre de Recherche sur l'inflammation UMR 1149, Paris, France; ^7^ Pediatric Department, University Hospital of Dijon, Dijon, France; ^8^ Department of Pediatrics, Hôpital Mère-Enfants, Nantes, France

**Keywords:** COVID-19, epidemiology, multisystem inflammatory syndrome in children, myocarditis, anakinra, interleukin-1

## Abstract

**Introduction:**

Multisystemic inflammatory syndrome in children (MIS-C) is a therapeutic emergency and can lead to myocardial dysfunction (17%–75%) and heart failure (52%–53%). Intravenous immunoglobulins (IVIG) and corticosteroids (CST) have been validated for the management of this condition. Recent reports suggest that an interleukin-1 (IL-1) receptor antagonist, namely anakinra, may be a valuable add-on to the 2019 novel coronavirus disease (COVID-19) treatment for refractory patients. The purpose of this study was to describe the clinico-biological characteristics of patients treated with anakinra as well as the efficacy and safety of subcutaneous anakinra therapy in this condition.

**Methods:**

The prospective multicentre study of children hospitalized for MIS-C between March 2020 and September 2022, including 23 international paediatric centres, followed for a mean duration of 3.072 ± 3.508 months. The patient data were extracted from the Juvenile Inflammatory Rheumatism (JIR) cohort. The clinico-pathological characteristics, cardiac ultrasound data, and adverse events were reported in patients receiving anakinra.

**Results:**

Of the 470 children admitted with MIS-C, 18 French patients (50% girls) with a mean age of 10.06 ± 3.9 years were treated with subcutaneous anakinra. Anakinra was used in two situations, macrophage activation syndrome (MAS) (4 patients) and heart failure (14 patients) with a median left ventricular ejection fraction (LVEF) of 39.5% (30%–45%). The average dose of anakinra received was 2.53 ± 1.3 mg/kg/day for a median duration of 3 days. Prior to introduction, 78% (*n* = 14/18) of the patients had received CST and 56% (*n* = 10/18) had received IVIG. Only two patients received IVIG alone and six received CST alone plus anakinra. In 10% of cases, IVIG was poorly tolerated from a cardiovascular point of view and was discontinued. Transient elevations in serum transaminases were noted in four patients on anakinra without the need for treatment or dose modification. In all patients, rapid (48 h) improvement in myocardial function was observed (LVEF > 55%) with a concomitant significant decrease in myocardial enzymes (*p* < 0.05). All patients survived with complete recovery of cardiac function without sequelae.

**Conclusions:**

Subcutaneous anakinra appears to be a safe and effective treatment for the management of heart failure or MAS in MIS-C patients. The value of IVIG in these two situations remains to be reviewed.

## 1 Introduction

Multisystemic inflammatory syndrome in children (MIS-C) is a therapeutic emergency and can lead to myocardial dysfunction (17%–75%) (1, 2) and heart failure in up to 52%–53% of the cases (3). The first cases in France have appeared with a median delay of 4 weeks after the first wave of the COVID-19 pandemic in March 2020 (4). Being a previously unknown disease, the management of MIS-C initially followed the model of Kawasaki disease (KD), a condition to which it seemed to be related. Therefore, intravenous immunoglobulins (IVIG) and corticosteroids (CST) have been massively used for alleviating systemic inflammation (5). Finally, the association of both IVIG and CST demonstrated effectiveness in association with inotropic drugs for critically ill patients (6, 7). However, on the practical level, the use of IVIG has had three main limitations. First, patients with advanced heart failure were unable to receive them or only partially, because of their high-volume load. Second, some patients remained refractory to this treatment strategy with irreversible chock syndrome. Third, the massive demand for IVIG at a given period has gradually led to difficulties in monitoring their production with a very marked drop in their availability.

For these reasons, an alternative or add-on treatment with subcutaneous anakinra, an interleukin-1(IL-1) receptor antagonist blocking both the IL-1α and the IL-1β, was progressively entered in our treatment strategy for MIS-C-related shock syndrome. IL-1 is a pro-inflammatory cytokine known to be central to the development of cardiac inflammation in various situations like pericarditis and myocarditis, and also KD-related coronary aneurysms (8–10). The IL-1α precursor is constitutively present in endothelial cells. It has also been found in endothelial membrane fragments, known as “apoptotic bodies,” which are released in the event of vascular inflammation and cause neutrophilic inflammation (11). In addition, in case of injury of the myocardial tissue, the dying cells release IL-1α, which is sensed as a danger signal that activates the inflammasome with processing and release of active IL-1β (12). The release of IL-1β induces the death of cardiomyocytes resulting in heart failure. There is an increasing number of reports and clinical trials exploring the efficacy of IL-1 blockade, essentially with the IL-1 receptor antagonist anakinra (blocking both the IL-1α and the IL-1β), for recurrent auto-inflammatory pericarditis, acute myocarditis with shock, and KD-related cardiac complications (13–15).

Our primary objective was to describe the clinico-biological characteristics of patients treated with anakinra in France prospectively included in the Juvenile Inflammatory Rheumatism (JIR) cohort, and to compare them with patients with MIS-C who did not receive anakinra. Our secondary objective was to study the efficacy and tolerability of anakinra on heart failure [normalization of cardiac ultrasound, left ventricular ejection fraction (LVEF), and cardiac markers when available] in patients with MIS-C.

### 1.2 Patients and methods

The JIR cohort (https://www.jircohorte.org) is an international prospective and retrospective cohort of patients with paediatric inflammatory rheumatism (clinical trial: NCT02377245) (16). Between March 2020 and September 2022, French centres prospectively recorded data on their patients with a diagnosis of MIS-C. The database includes demographic data, vital sign measurements, and diagnostic and treatment information for all patients.

We collected all cases of French MIS-C entered by referring physicians during the study period. Then, we selected all patients meeting the World Health Organization’s diagnostic criteria for MIS-C (17). Among them we identified patients who had received at least one injection of subcutaneous anakinra for an MIS-C-related event. We collected epidemiological data, disease characteristics, and biological and cardiac ultrasound data of the entire cohort of patients.

### 1.3 Anakinra group

We described the clinico-biological characteristics of patients treated with anakinra, their cardiological data, treatments received prior to anakinra, as well as their adverse effects and total length of hospitalization in this population. We then compared these data with that of patients with MIS-C who had not received anakinra, to understand the reasons for using anakinra.

Secondly, we looked at the evolution of cardiac markers (echographic and biological) in patients 48 h before and 48 h after anakinra treatment, to evaluate the response to treatment.

Finally, we looked at the tolerability of anakinra. Adverse events were defined as any undesirable or suspected reaction that occurred after anakinra treatment. Liver enzymes were accepted as increased if the elevation was ≥2 times the upper limit of normal.

### 1.4 Ethical considerations

All patients included in the JIR cohort received prior information on the study and were not opposed to having their medical data collected. This study was compliant with the principles of the Declaration of Helsinki, and the JIR cohort was approved by the French ethics committee Comité Consultatif sur le Traitement de l’Information en matière de Recherche dans le domaine de la Santé (CCTIRS) on 21 April 2015. The electronic case report form was approved by the Commission Nationale de l'Informatique et des Libertés (CNIL).

### 1.5 Statistical analyses

Numeric variables were expressed as median and discrete outcomes as absolute and relative (%) frequencies. We created two groups according to the values “il1_treatment.” Group comparability was assessed by comparing baseline demographic data and follow-up duration between groups. Normality and heteroscedasticity of continuous data were assessed with the Shapiro–Wilk and Levene's tests, respectively. Continuous outcomes were compared with unpaired Student’s *t*-test, Welch *t*-test, or Mann–Whitney *U*-test according to data distribution. Discrete outcomes were compared with *χ*^2^ or Fisher's exact test accordingly. The alpha risk was set to 5% and two-tailed tests were used. Statistical analysis was performed with EasyMedStat (version 3.24; www.easymedstat.com).

## 3 Results

### 3.1 Whole cohort

**Figure 1:**
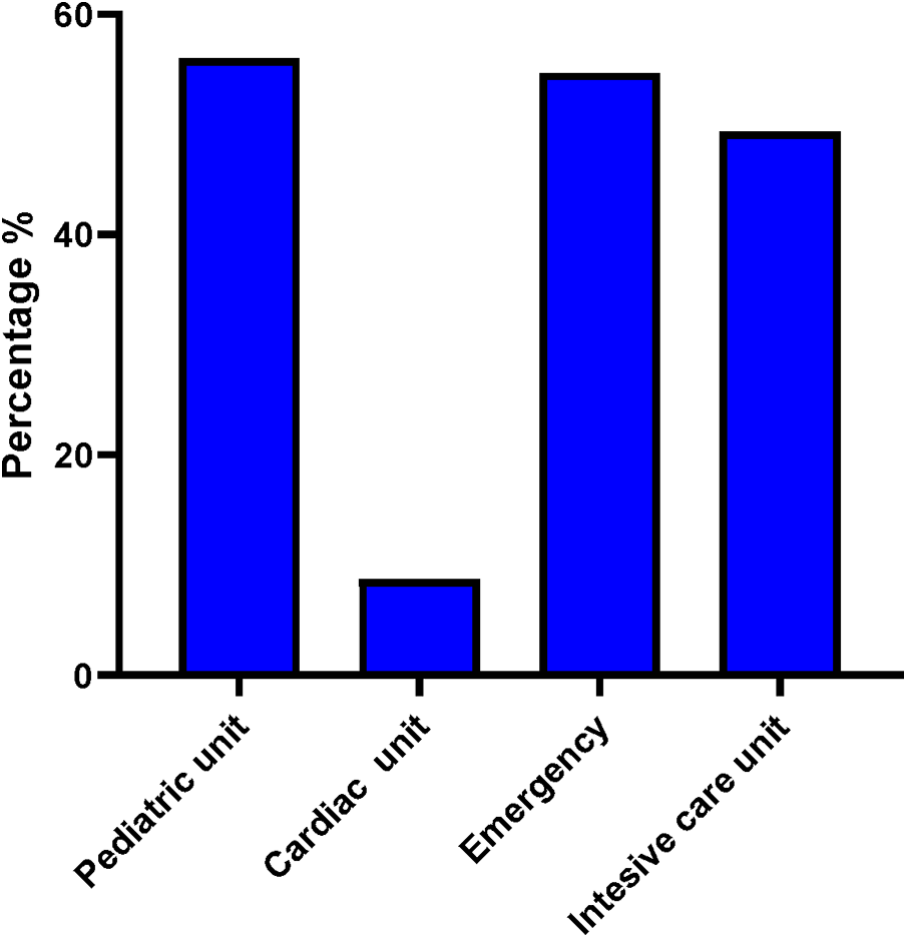
Primary care unit for patients with MIS-C (*n* = 470).

We included 470 children admitted with MIS-C (Table 1) from 30 different French centres. Among them, 276 were boys and 194 were girls (SR: 1.43). The respective distribution of ethnicities (*n* = 116) showed the following: Western Caucasians (85.34%), North Africans (4.31%), black Africans (7.76%), and Asians (0.86%). Their median age at onset of MIS-C was of 8.82 years (0.05–17.55) and their median age at inclusion was of 9 years (0.05–18). The median duration of fever was of 6 days (1.00–12.00). The signs related to MIS-C included, by order of frequency, deterioration of the general condition (93.99%, *n* = 333), digestive manifestations (82.7%, *n* = 370), cardiac manifestations (81.36%, *n* = 338), cutaneous rash (78.51%, *n* = 363), and irritability (45.67%, *n* = 208). More than half of the patients required intensive care referral for cardiac rescue or other MIS-C-related complication (Figure 1). During hospitalization between day 0 and discharge, 75% (292/387) of the patients received IVIG and 73% (283/387) received either intravenous or oral corticosteroids. A total of 18 patients received anakinra, 2 with IgIV and 2 alone.

**Table 1:** Description of the main clinical and biological parameters of the 470 patients with MIS-C during hospitalization (initial flare) and the different treatments used.

	Any treatment	IVIG only	VIG and glucocorticoids	Glucocorticoids only	IVIG, glucocorticoids and a biologic	Glucocorticoids and a biologic
	(*N* = 470)	(*N* = 57)	(*N* = 223)	(*N* = 50)	(*N* = 8)	(*N* = 6)
Demographic characteristics						
Male sex—no. (%)	276 (58.7)	29 (51)	136 (61.0)	28 (56.0%)	4 (50)	3 (50)
Age—year						
Median (IQR)	8.87 (5.2–11.7)	7.5 (3.9–11.1)	7.46 (3.9–11.1)	9.96 (6.04–11.96)	9.0 (5.9–13.5)	10.2 (6.7–10.6)
Range	0.1–24.4	0.3–17.0	0.3–17	1.6–17.6	0.9–20.9	3.6–12.8
Clinical and cardiac characteristics						
Troponin level max						
No. of patients	256	33	173	30	6	3
Median (IQR)—ng/L	95 (28.0–524.0)	646 (189–36,000)	89 (27.0–386.0)	52.5 (18.5–228.7)	90.5 (66–112)	95 (55–114)
B-type natriuretic peptide level max						
No. of patients	11	5	6	NC	NC	NC
Median (IQR)—ng/L	26,597.0 (2,230–207,9100)	26,597 (2,600–63 141)	20,269 (977.7–113,680)			
N-terminal pro–B-type natriuretic peptide level max						
No. of patients	199	24	129	28	6	5
Median (IQR)—ng/L	4,135 (1,677–13,245)	5,507 (2,036–30,133)	4,068 (1,600–10,478)	4,030.5 (1,519–13,811.5)	18,832 (4,830–20,482)	7,444 (5,016–9,735)
LVEF <55% during hospitalization—no. (total)	27 (46)		19 (33)	1 (3)	3 (5)	3 (5)
Interventions						
Vasopressors—no./total (%)	7/385 (1.8)	6/57 (10.5)	34/223 (15.2)	1/49 (2)	0/8 (0)	0/6 (0)
Extracorporeal membrane oxygenation—no./total (%)	1/60 (1.7)	0/21 (0)	1/31 (3.2)	2/2 (100)	0/2 (0)	0/2 (0)
Discharged alive—no. (%)	470 (100)	57 (100)	223 (100)	50 (100)	8 (100)	6 (100)
Median hospital length of stay among survivors (IQR)—days	7 (5.2–10.0)	8 (6–12)	7 (5–10)	8.0 (4.2–10.0)	13 (10.7–16.2)	9.5 (9–12.2)
Died—no. (%)	0 (0)	0 (0)	0	0 (0)	0 (0)	0 (0) A12:A7:G25

LVEF, left ventricular ejection function; Max, maximum value during hospitalization.

Four patients received anakinra alone or after IVIG but without corticosteroids. These four patients were not represented.

### 3.2 Cohort receiving anakinra

Eighteen patients (50% girls) with a median age of 8.85 (0.01–24.42) years at diagnosis were treated with subcutaneous anakinra and monitored for a median of 3.99 months (0.06–12.1). Anakinra was used in seven French centres in patients considered to have a severe form of MIS-C either with macrophage activation syndrome (MAS) (4 patients) or heart failure (14 patients) with a median LVEF of 39.5% (30%–45%). The average dose of anakinra received was 2.53 ± 1.3 mg/kg/day for a median duration of 3 days (1–70). Prior to the introduction, 77.782% (*n* = 14/18) of patients had received corticosteroids and 55.56% (*n* = 10/18) had received IVIG. Only two patients had IVIG alone and six had corticosteroids alone in addition to anakinra (including the four patients with MAS) (Table 2). Two patients received only anakinra without corticosteroids or IVIG. In 40% of the cases, IVIG was poorly tolerated with worsening of cardiac function and was discontinued. Transient elevations in serum transaminases (twice the normal level) were noted in four patients on anakinra with no need for change in treatment or dose.

**Table 2:** Comparison of demographic, clinical characteristics, and treatments of patients at the time of flare between those who received or did not receive anakinra.

Variable	No Anakinra	Anakinra	*p*-Value
	*N* = 367	*N* = 18	
Gender			
Male	210 (57.22%)	9 (50.0%)	0.629
Women	157 (42.78%)	9 (50.0%)	
	*N* = 367	*N* = 18	
Age at first symptoms (years)	8.56 (± 4.44)	10.06 (± 3.97)	0.168
	*N* = 365	*N* = 18	
Ethnicity			
african_west_indies	1 (0.88%)	0 (0.0%)	0.587
asia	3 (2.63%)	0 (0.0%)	
caucasians	34 (29.82%)	1 (25.0%)	
mediterranean_basin	30 (26.32%)	3 (75.0%)	
mixed	9 (7.89%)	0 (0.0%)	
north_central_america	2 (1.75%)	0 (0.0%)	
sub_saharan_africa	30 (26.32%)	0 (0.0%)	
unknown	5 (4.39%)	0 (0.0%)	
	*N* = 114	*N* = 4	
Total hospitalization time (days)	9.38 (± 19.58)	17.24 (± 16.91)	**<0** **.** **001**
	*N* = 365	*N* = 17	
Symptoms of initial acute flare			
Irritability	79 (42.25%)	10 (90.91%)	**0** **.** **03**
Yes	108 (57.75%)	1 (9.09%)	
No	*N* = 187	*N* = 11	
Deterioration of general condition	283 (94.33%)	15 (100.0%)	>0.999
Yes	17 (5.67%)	0 (0.0%)	
No	*N* = 300	*N* = 15	
Musculoskeletal manifestation	62 (22.55%)	5 (33.33%)	0.349
Yes	213 (77.45%)	10 (66.67%)	
No	*N* = 275	*N* = 15	
Cutaneous manifestation	253 (77.85%)	15 (88.24%)	0.544
Yes	72 (22.15%)	2 (11.76%)	
No	*N* = 325	*N* = 17	
Respiratory manifestation	99 (34.74%)	8 (53.33%)	0.17
Yes	186 (65.26%)	7 (46.67%)	
No	*N* = 285	*N* = 15	
Digestive manifestation	275 (83.08%)	14 (82.35%)	>0.999
Yes	56 (16.92%)	3 (17.65%)	
No	*N* = 331	*N* = 17	
Ocular manifestation	178 (59.73%)	11 (64.71%)	0.802
Yes	120 (40.27%)	6 (35.29%)	
No	*N* = 298	*N* = 17	
Vascular manifestation	10 (3.65%)	2 (13.33%)	0.123
Yes	264 (96.35%)	13 (86.67%)	
No	*N* = 274	*N* = 15	
Cardiac manifestation	247 (82.33%)	15 (93.75%)	0.324
Yes	53 (17.67%)	1 (6.25%)	
No	*N* = 300	*N* = 16	
Initial CRP (mg/L)	124.28 (± 94.89)	176.83 (± 92.1)	0.08
	*N* = 99	*N* = 10	
CRP max (mg/L)	235.76 (± 104.78)	257.31 (± 125.14)	0.606
	*N* = 224	*N* = 10	
ALAT max (UI/L)	68.42 (± 71.68)	85.06 (± 59.97)	0.118
	*N* = 204	*N* = 13	
ASAT max (UI/L)	84.16 (± 88.92)	69.08 (± 67.1)	0.358
	*N* = 202	*N* = 12	
Ferritin max (µg/L)	401,418.06 (± 985,141.88)	417,654.45 (± 644,059.57)	0.419
	*N* = 186	*N* = 11	
Nt-proBNP max (ng/L)	16,827.39 (± 88,506.54)	13,961.54 (± 15,906.99)	0.229
	*N* = 186	*N* = 13	
Troponine max (ng/L)	17,66,726.92 (± 25,653,463.86)	142.73 (± 167.67)	0.582
	*N* = 246	*N* = 10	
Ferritin max (µg/L)			
LVEF (%)	50.55 (± 12.6)	43.0 (± 10.95)	0.208
	95% CI: (46.57; 54.53)	95% CI: (29.4; 56.6)	
	*N* = 41	*N* = 5	
Corticotherapy treatment	267 (72.75%)	14 (77.78%)	0.789
Yes	100 (27.25%)	4 (22.22%)	
No	*N* = 367	*N* = 18	
Total corticotherapy duration (days)	5.79 (± 4.1)	10.64 (± 6.01)	**<0** **.** **001**
	*N* = 367	*N* = 14	
IVIG treatment			
Yes	280 (76.29%)	10 (55.56%)	0.088
No	87 (23.71%)	8 (44.44%)	
	*N* = 367	*N* = 18	
IVIG adverse event			
Yes	0 (0%)	1 (10%)	**0** **.** **034**
No	280 (100%)	9 (90%)	
	*N* = 280	*N* = 10	
Beta-blocker treatment			
Yes	13 (3.54%)	3 (16.67%)	**0** **.** **033**
No	354 (96.4%)	15 (83.33%)	
	*N* = 367	*N* = 18	

Bold values denote statistical significance (*p* < 0.05).

**Figure 2:**
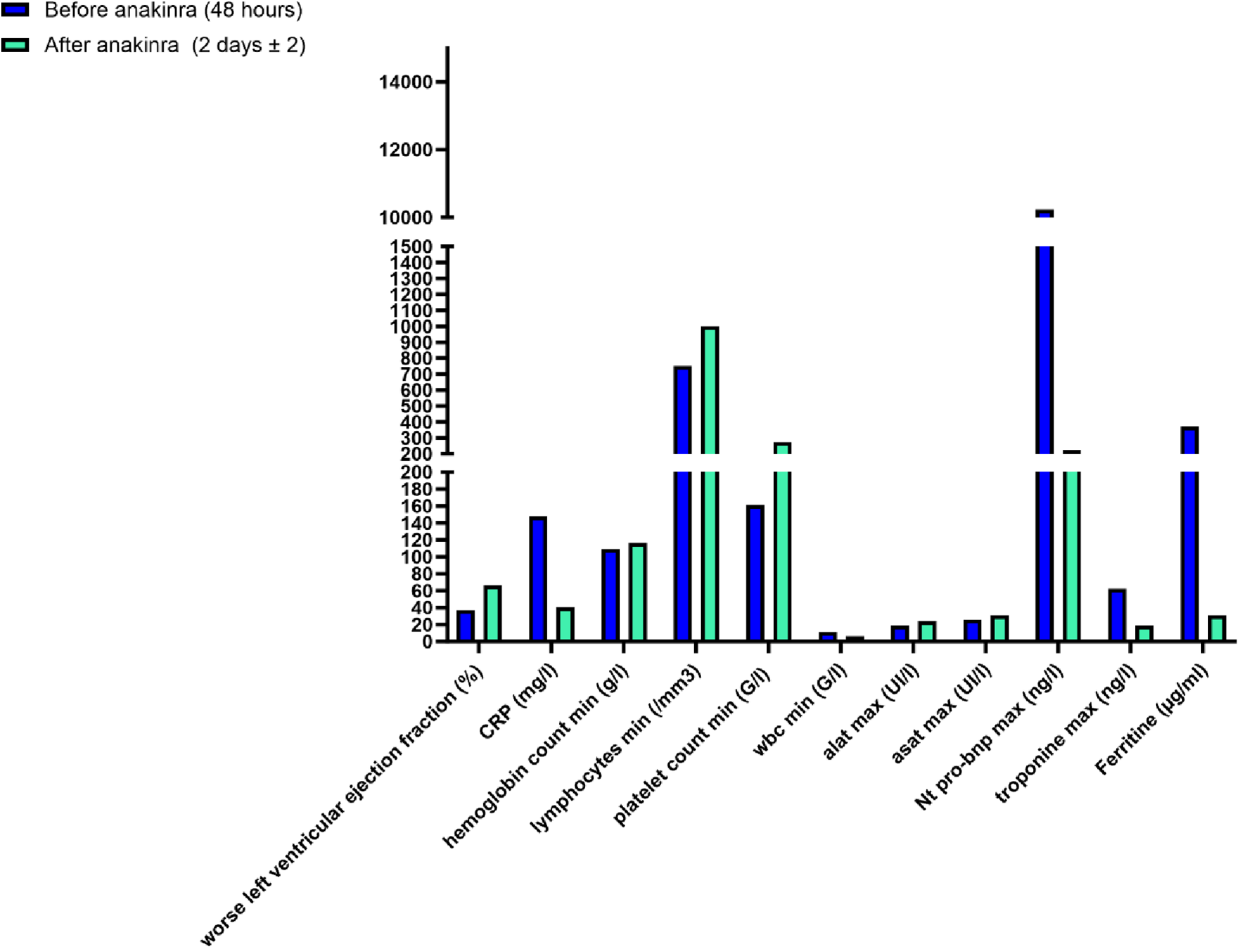
Biological and cardiac evolution of patients with MIS-C after 2 ± 2 days of anakinra.

In all patients, rapid (48 h) improvement in myocardial function was observed (LVEF > 55%). In addition to normalization of cardiac ultrasound, a concomitant significant decrease in myocardial enzymes (Nt-proBNP decreased from a mean of 10.464 to 1.480 ng/L and troponin from a mean of 70.14 to 31.5 ng/L in 3 days) and C-reactive protein (CRP) (from a mean of 146 to 43 mg/L in 2.8 days) when available (*n* = 8/17) (*p* < 0.05) was noted. All patients survived with complete recovery of cardiac function without sequelae (Figure 2).

No side effects have been reported with anakinra (neither injection site reactions nor hepatitis). Only four patients experienced transient elevations in serum transaminases (max three times normal), which did not lead to reduction or discontinuation of treatment.

### 3.3 Comparison between patients with MIS-C receiving and not receiving anakinra

Both the groups were similar regarding the majority of features related to MIS-C (Table 2). However, we found that patients receiving anakinra tended to have more days of fever, and had significantly more irritability (*p* < 0.006), more vascular features (*p* = 0.005), and MAS (*p* < 0.001). The biological variables associated with the use of anakinra were lower level of haemoglobin (120.44 vs. 68.52 g/L) and higher duration of corticosteroid treatment (mean 5.79 ± 4.1 vs. 10.64 ± 6.01, *p* < 0.001). Patients treated with anakinra tended to have received less IVIG than those who did not (55.6% vs. 76%), although this was not significant in our analyses (*p* = 0.088).

## 4 Discussion

Our study reflects the management of MIS-C in France. The use of anakinra ultimately remained fairly marginal (3.8% of cases), but conversely the reasons for resorting to it appeared quite clear in our study, which were essentially shock syndrome, resistant MIS-C, and episodes of macrophage activation syndrome.

The use of anakinra was guided by the experience of paediatricians who used it in similar situations, such as the refractory Kawasaki syndrome (13) and MAS of auto-inflammatory origin (18). This practice was also encouraged by evidence of the role of IL-1 in COVID-19 post-vaccine myocarditis (19), and anakinra was included in the list of possible therapies as of March 2020 (20). Anakinra, a recombinant IL-1 receptor antagonist, is fast-acting and very safe, thanks to its very short half-life. Interestingly, the onset of action of anakinra in heart failure was remarkably short, resulting in rapid correction of LVEF. In addition, the level of Nt-proBNP, a marker of myocardial fibre stretch, fell strikingly under anakinra treatment. The impression of an on/off effect of anakinra on shock also very clearly reflects the similarity of MIS-C to the toxic shock syndrome previously known to be secondary to staphylococcal infections. In line with our observation, the duration of anakinra treatment was short, and effective doses remained relatively low: 1–3 mg/kg compared with the American College of Rheumatology (ACR) 2022 recommendations (>4 mg/kg/day IV or SC) (21) or the Italian recommendations (8 mg/kg/day IV or 4–6 mg/kg/day SC) (22) but closer to the Turquish study (2.7 mg/kg/day) (23). Intravenous anakinra was not used in France because of the lack of awareness of this route of administration. Two patients did not receive any IVIG despite the recommendations. This therapeutic attitude has also been applied in certain centres in Europe, particularly in Italy, given the cardiac failure of patients with MIS-C (24). All patients who had experienced a cardiogenic shock situation had a complete and definitive recovery without sequelae (data not shown).

Injection site reaction is the most common side effect of anakinra treatment (21). However, this side effect has not been reported, as mentioned in the literature (23, 25). This discrepancy may be linked to the fact that patients are warned of this expected impressive but benign effect, and are usually prescribed an anti-histamine or hydrocortisone cream to apply after the injection in case of local reaction. This may have trivialized the effect for parents and/or children who did not see fit to report it. Transaminase elevation is another complication that has been described and found in patients with MIS-C [more than three times the upper limit of normal in 13% (*n* = 12) of patients in the Cavalli et al. study (25) and 15.8% (*n* = 13) in the Çaǧlayan et al. study (23)]. However, this elevation was not significant in our patients and it is difficult to clearly state whether this was an adverse effect or was related to the disease course.

Even prospective and collected homogeneously, our study is a real-life observational study rather than a clinical trial. Therefore, we can absolutely not conclude from this experiment that anakinra alone was the critical element in the reversibility of cardiac failure. The available data in the literature are unfortunately not much more enlightening on this subject. Indeed, a number of case reports and case series mentioning the use of anakinra in MIS-C have been published during the pandemic and a review of 41 papers appeared in 2021 (26). From these publications, it appeared that the reason for using anakinra was not always or uniformly reported, nor were the doses, the durations, and the responses to this treatment, even if they could be approximated to over 90%. As these studies were essentially case reports describing efficacy, and because patients received a number of concomitant treatments, the beneficial effect of anakinra by itself or by add-on effect with either corticosteroids or IVIG or both could not be determined. In our study, only two adolescents received only anakinra: one with MAS and the other who was treated for Still’s disease before being labelled MIS-C because of hyperferritinaemia. A retrospective cohort study in a US surveillance registry compared MIS-C cases in patients receiving IVIG and glucocorticoids vs. anakinra plus IVIG and/or glucocorticoids on days 0–1 (first calendar day of immunomodulator treatment). The global use of anakinra was 13% (193/1,516). They did not find any differences among patients receiving or not receiving anakinra in terms of vasopressor requirement and drop of the CRP (27). Recently, Taddio et al. in Italy showed that early treatment with anakinra is safe and very effective in patients with severe MIS-C as in our cohort with or without IVIG (28).

Our study has several limitations, non-homogeneous care, dependent on the experience of the centres. Indeed, the use of anakinra reflects the experience of a few French centres with expertise in the use of anti-IL-1 drugs. In fact, only 7 out of 30 centres used it, and among them, one centre treated 45% of patients and another, 17% of patients. In addition, we can note a selection bias since only French centres participating in the JIR cohort are represented in this study. Probably other centres have used anakinra in France but were not taken into account in this study. Finally, the number of missing data is another limitation of the interpretation. Only some data were collected in the database from hospitalization reports. Biological examinations were therefore often lacking. Likewise, the LVEF value was not always reported. Nevertheless we estimated the completeness of the entries in the database at approximately 34% if we refer to the 1,092 cases of MIS-C declared to Public Health France over all the waves (29). We are therefore fairly confident that the data published globally reflected the practice applied to all the French cases. We also know that these may have evolved over the different waves with a greater use of corticosteroids during the second compared with the first (30).

## 5 Conclusion

Our prospective study has shown that subcutaneous anakinra was safe and rapidly effective for reversing heart failure or MAS in IVIG and corticosteroid unresponsive, refractory MIS-C cases.

## Data Availability

The datasets presented in this article are not readily available because requests to access them must be made to the Juvenile Inflammatory Rheumatism (JIR) Cohort. Requests to access the datasets should be directed to Francois Hofer, francois.hofer@jircohorte.ch.
